# Predetermined Change Control Plans: Guiding Principles for Advancing Safe, Effective, and High-Quality AI-ML Technologies

**DOI:** 10.2196/76854

**Published:** 2025-10-31

**Authors:** Eduardo Carvalho, Miguel Mascarenhas, Francisca Pinheiro, Ricardo Correia, Sandra Balseiro, Guilherme Barbosa, Ana Guerra, Dulce Oliveira, Rita Moura, André Martins dos Santos, Nilza Ramião

**Affiliations:** 1 Institute of Science and Innovation in Mechanical and Industrial Engineering (INEGI), Campus da FEUP Rua Dr. Roberto Frias, 400, Porto, 4200-465, Portugal, +351 229578710; 2 Faculty of Medicine, Universidade do Porto (FMUP), Porto, Portugal; 3 Hospital de São João, Porto, Portugal; 4 University of South Alabama, Mobile, AL, United States; 5 BioGHP, Lisboa, Portugal; 6 SmartMDR, Coimbra, Portugal

**Keywords:** predetermined change control plans, artificial intelligence, health care, machine learning, regulation

## Abstract

The adaptive nature of artificial intelligence (AI), with its ability to improve performance through continuous learning, offers substantial benefits across various sectors. However, current regulatory frameworks are not intended to accommodate this adaptive nature, and they have prolonged approval timelines, sometimes exceeding one year for some AI-enabled devices. This creates significant challenges for manufacturers who must deal with lengthy waits and submit multiple approval requests for AI-enabled device software functions as they are updated. In response, regulatory agencies like the US Food and Drug Administration (FDA) have introduced guidelines to better support the approval process for continuously evolving AI technologies. This article explores the FDA’s concept of predetermined change control plans and how they can streamline regulatory oversight by reducing the need for repeated approvals, while ensuring safety and compliance. This can help reduce the burden for regulatory bodies and decrease waiting times for approval decisions, therefore fostering innovation, increasing market uptake, and exploiting the benefits of artificial intelligence and machine learning technologies.

## Introduction

The use of artificial intelligence (AI) and machine learning (ML) technologies is growing exponentially across industries, and the amount of data gathered in health care and other sectors increases the impact and importance of such technologies as well as the need for regulatory frameworks that accommodate continuous learning. In health care, AI-ML technologies are being used to assist health care providers, aid diagnosis, personalize treatments, predict health problems, and improve patient care. One of the biggest advantages of AI-ML in software is its ability to improve performance by learning from new data and real-world use and experience. However, these technologies pose some unique challenges imposed by the dynamic learning typically involved in the development, deployment, use, and maintenance of such technologies. They are developed iteratively, which means that the software changes are continually applied, either manually or automatically [1].

The large majority of the health care tools that use AI-ML technologies are classified by most jurisdictions as software as a medical device (SaMD), which falls under stringent regulatory oversight. In the United States, the FDA categorizes SaMD based on risk levels (Class I, II, and III), determining the appropriate approval pathway, either the premarket approval (PMA), the 510(k), or De Novo classification. The FDA approved 95,147 devices up to the end of 2024 with the 510(k) or the De Novo pathway and a total of 1678 devices via the PMA pathway. Within the PMA pathway, a total of 53,315 PMA supplements were submitted, with an average of 3177 supplements per device. These supplements are documents required for a change affecting the safety or effectiveness of a device with an approved PMA. This highlights the need for mechanisms to update devices while ensuring safety and effectiveness and with less burden for manufacturers and regulators, which is particularly relevant for devices with AI-ML technologies.

Traditional regulations were not intended to accommodate the adaptive nature of AI-enabled device software functions (AI-DSFs), which involve continuous learning strategies that affect the clinical safety, performance, and benefit of the device. The regulations state that devices should be used in their approved form, without subsequent changes that affect their functionality, which means that AI-DSFs could not get approval because they change dynamically without manufacturer supervision and, as a result, their updated state no longer conforms to the approved and tested state. If AI-DSFs do not change after market approval, there will not be a problem. However, this limits the benefits of continuous learning systems that can improve their quality and performance over time. Traditional regulations require devices to be “locked” upon approval or to submit a new submission for approval after modifications. These hinder continuous improvement and adaptation to new environments, often resulting in performance degradation when the device encounters scenarios that differ from those used for training the AI-ML technology. Consequently, data collected in the meantime is unused, limiting opportunities to enhance accuracy, safety, and effectiveness [2].

This problem raised the attention of jurisdictions that are developing novel, faster, and robust methods for devices that change after being placed on the market. In 2019, the FDA published a paper describing a potential approach to premarket review for AI-ML-driven software modifications. In this paper, a framework for modifications to AI-ML-based SaMD was proposed, promoting a mechanism for manufacturers to continually maintain the safety and effectiveness of SaMD [3].

**Figure 1.**
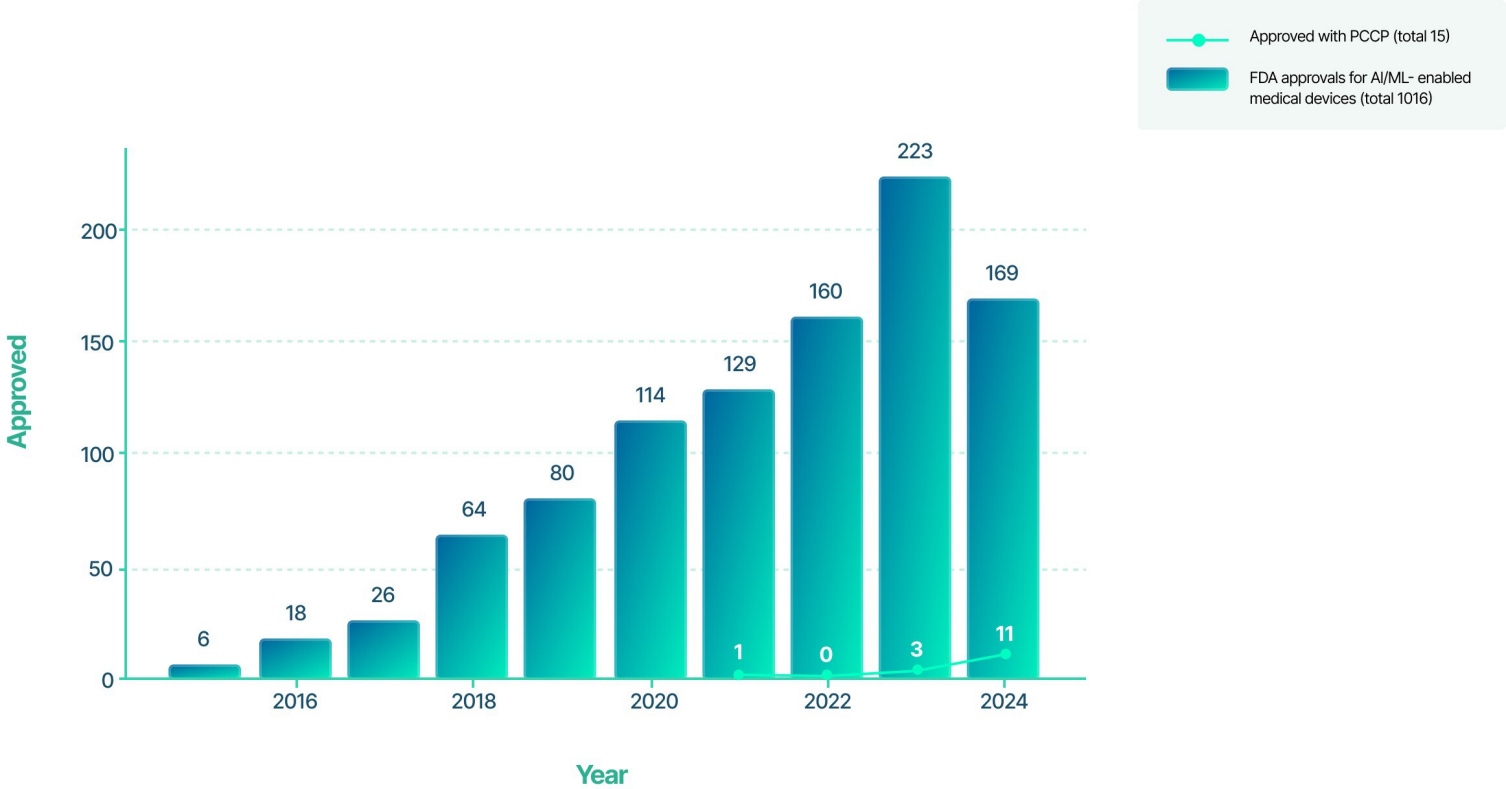
Artificial intelligence and machine learning-enabled medical devices approved by Food and Drug Administration over time. AI: artificial intelligence; FDA: Food and Drug Administration; ML: machine learning; PCCP: predetermined change control plan.

Afterward, in 2021, the FDA issued a document with guiding principles for the development of medical devices with machine learning (ML) [4]. Later, in 2023, FDA, UK Medicines and Health Products Regulatory Agency, and Health Canada published 5 guiding principles for predetermined change control plans (PCCPs) in ML-enabled medical devices to ensure safety and effectiveness throughout the device’s total product lifecycle [5]. More recently, the FDA published the marketing submission recommendations for a PCCP for AI-DSF, which supports the iterative improvement of AI-enabled devices with reasonable safety and effectiveness without the need for additional marketing submissions to implement each of the modifications outlined in a PCCP [6]. Up until the end of 2024, a total of 1.016 AI-ML-enabled medical devices have been approved by the FDA [7], 53 devices with PCCPs [8], and 15 AI-ML-enabled medical devices with a PCCP (Figure 1).

In the European Union (EU), the Medical Device Regulation (MDR) defines a rigorous certification process, and there is awareness of the limitations of the current legislation, as recommendations to monitor significant modifications, namely changes that violate compliance with existing regulatory requirements or any change to the preapproved intended purpose, have been included in the AI Act [9,10].

The EU, similarly to the United States, also proposed a regulatory framework for AI systems, but it follows a stricter and more granular approach. The EU AI Act applies to all AI systems across multiple sectors, and it classifies AI systems into risk categories. AI-ML-enabled medical devices are considered high-risk and lead to more strict requirements. Providers of high-risk AI systems must implement a quality management system, where it is necessary to outline, for example, risk management strategies, data governance, technical documentation, and postmarket monitoring. The EU AI ACT also emphasizes postmarket surveillance and requires manufacturers to analyze how the AI system is performing after deployment. There is a clear focus on ensuring that high-risk systems have detailed documentation and plans for continuous monitoring, but there is a clear lack of guidance on how to deal with the changes to such devices and how to account for the adaptability of AI-ML technologies that are intended to continuously learn and change over time [11].

Changes predetermined by the manufacturer at the moment of initial conformity assessment should be defined and described, including technical solutions adopted to ensure continuous compliance of the AI system with the relevant requirement, but they shall not constitute a “substantial modification.” However, there is no clear definition of what is considered substantial, and contrary to the FDA, the EU AI Act lacks objectivity on what can be accommodated as a modification without the need for repeated assessment of conformity [11]. In a question and answer document provided by the EU, it is stated: “This assessment has to be repeated if the system or its purpose are substantially modified.” [12], which emphasizes the ambiguity and vagueness. The current regulatory framework still lacks detail on implementing algorithm change protocols, and even though the importance of postmarket performance monitoring is reflected in the EU AI Act, there are no specific guidelines [13]. This limitation is recognized and in Article 96 of the EU AI Act, where it states that the commission should develop guidelines on the practical implementation of the provisions related to substantial modification [11].

The key differences between the regulation of AI-ML medical devices in the United States and the EU are summarized in Table 1.

**Table 1.** Differences in AI^a^-ML^b^ medical device regulation in the European Union (EU) and United States (US).

Regulation Aspect	European Union	United States
Regulatory bodies	Medical device regulation (MDR) and EU AI Act	FDA^c^
Approval pathways	CE^d^ marking	510(k), De Novo, or PMA^e^
Risk classification	High risk	No explicit risk-based classification
Bias mitigation	Emphasis on fairness and transparency	Focus on diverse training data
Adaptability	Updates may require new conformity assessments and certifications	Allows iterative AI-ML model updates without full resubmission with PCCPs^f^
Postmarket monitoring	Strong emphasis with AI Act requiring continuous monitoring and reporting	FDA encourages postmarket surveillance and real-world performance monitoring

a AI: artificial intelligence.

b ML: machine learning.

c FDA: Food and Drug Administration.

d CE: Conformité Européenne.

e PMA: premarket approval.

f PCCP: predetermined change control plan.

Despite ongoing regulatory efforts, significant uncertainty remains regarding how to address continuous learning AI-ML systems and their intrinsic modifications and which regulations and legislation manufacturers should look at. To address this, this paper aims to explore the challenges and opportunities associated with regulating the adaptive features of AI and to explore how PCCPs can guide the iterative development of AI-ML systems while ensuring safety, effectiveness, and quality. The paper explains the key concepts of PCCPs and outlines the guiding principles of PCCPs in order to establish its foundations. Afterward, it discusses the implications of PCCPs and presents reference case studies. Finally, it identifies ongoing challenges and proposes future directions.

## Understanding PCCPs

PCCPs are regulatory mechanisms that allow manufacturers to implement preapproved modifications to systems or devices after market authorization while maintaining compliance with safety and performance standards.

**Figure 2.**
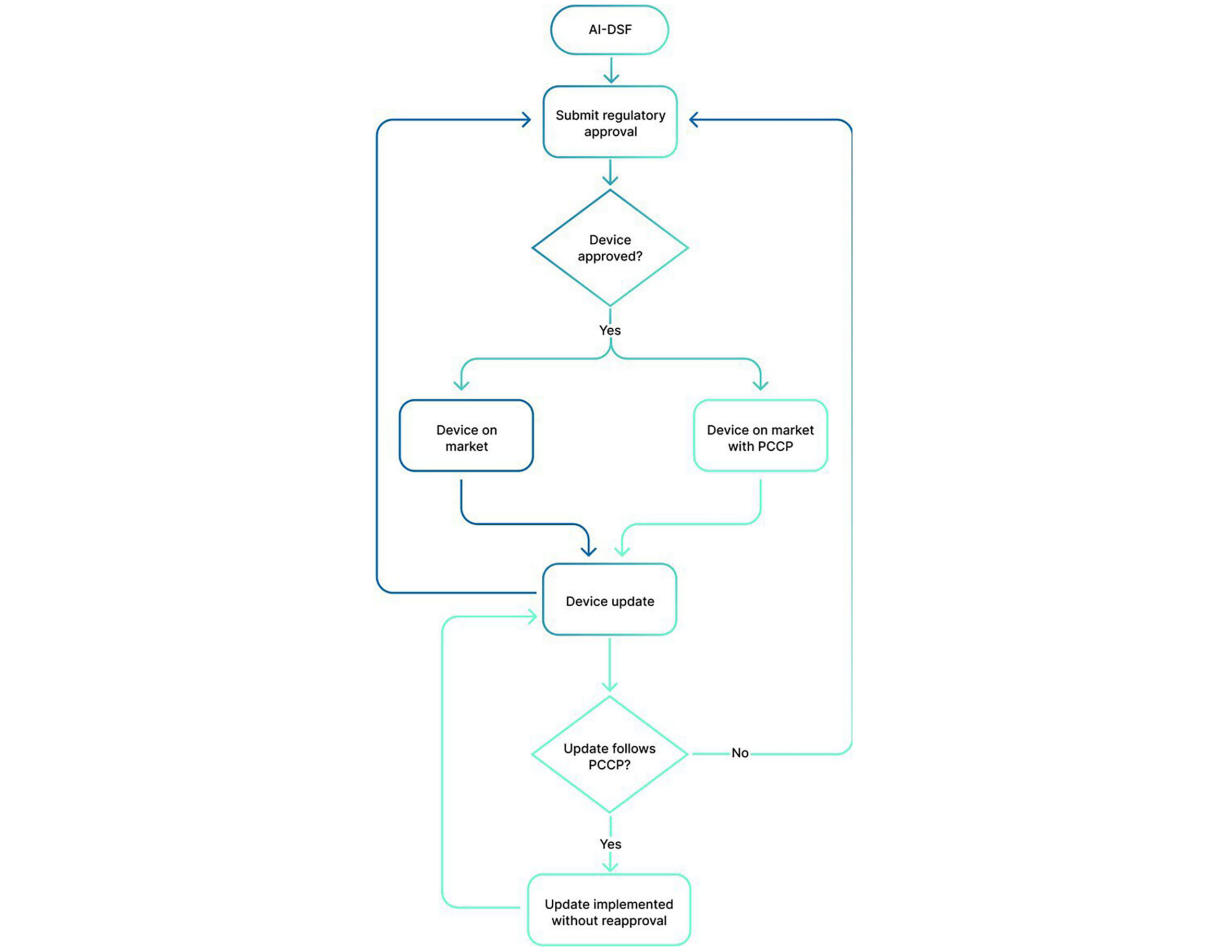
Regulatory approval process: traditional - blue; with a PCCP predetermined change control plan - green. AI-DSF: AI-enabled device software function; PCCP: predetermined change control plan.

Contrary to traditional control mechanisms, which assume that systems remain unchanged and are used in their approved form, PCCPs take into account the dynamic nature of AI-ML systems and exploit the benefits of continuous learning systems [14]. Traditional static validation methods for medical devices and AI-driven software rely on a fixed approval process, assuming that the system remains unchanged throughout its lifecycle. Nonetheless, this approach is insufficient for AI-ML systems due to their inherently dynamic nature. Unlike conventional software, AI-ML models evolve as they are exposed to new data, necessitating a regulatory approach that accommodates ongoing modifications while ensuring compliance and safety [15]. In this manner, PCCPs provide a structured regulatory mechanism that enables manufacturers to implement controlled AI-ML model updates without requiring extensive reapproval procedures. This adaptability allows manufacturers to refine AI-ML models through real-world data, mitigating risks associated with outdated static validation frameworks and clinical insights, leading to enhanced performance and improved outcomes. By preapproving modifications, PCPPs reduce the need for manufacturers to resubmit devices for approval after every update, leading to fewer inefficiencies and delays and faster time-to-market [2,10]. Figure 2 shows the traditional regulatory approval process (in blue) and the one with a PCCP (in green).

By including PCCPs in market submissions, manufacturers can proactively outline and secure premarket authorization for specific device changes. This reduces the need for additional submissions and subsequent regulatory approval, provided the changes are consistent with the preapproved PCCP established during the device’s marketing authorization. The development of PCCPs can streamline the implementation of modifications, ensuring that updates meet established safety, efficacy, and quality standards without requiring repeated resubmissions [2,6]. PCCP encompasses some key aspects:

- Planned modifications: identification of software components subject to updates, such as model refinements and performance enhancements.
- Data lifecycle management: implementation of robust data management practices to maintain compliance and data integrity.
- Implementation and performance evaluation: establishment of clear metrics to validate modifications.

Manufacturers should focus on addressing the following 4 questions to ensure that PCCPs have the fundamental concepts:

- What are the planned changes?
- How will the data lifecycle be managed?
- How will the changes be performed?
- How will the performance of the model with the changes be evaluated?

To get approval for a device with a PCCP, the market submission must include a standalone PCCP section, outlining planned modifications, risk assessments, and validation procedures. The planned modifications should also be included and discussed in the cover letter of the marketing submission, in the device description, labeling, and other relevant sections required for safety and effectiveness assessment. In addition, the authorized PCCP should include 3 main sections [6]:

1. Description of modifications, including a detailed account of proposed changes and their rationale. It is recommended that these modifications be specific, validated, and verified. For example, it is important to define if the change will be implemented automatically, manually, or a combination of both. Distinguishing global from local changes is also an important point to clarify because, in local changes, the modifications are implemented differently on the market, based on specific characteristics. The modifications appropriate for inclusion in a PCCP are those intended to maintain or improve the safety or effectiveness of the device, and, for AI-DSFs, they usually fall into one of the following types:
  - Modifications related to quantitative measures, such as improvements in performance due to model retraining with new data;
  - Modifications related to device inputs and compatibility, such as adding new inputs or transforming them, updates of the compatible software or hardware, and device interoperability;
  - Modifications related to use and performance
2. Modification protocol, which should describe a structured approach for developing, validating, and implementing modifications, is outlined in the Description of Modifications section of the PCCP. In addition, it should also include verification and validation strategies for the modifications, with predefined acceptance criteria. It is relevant that this section includes 4 components:
  - Data management practices, to outline how new data will be collected, annotated, curated, stored, and used to train, tune, and test the AI-DSF; retraining practices, to identify processing steps that are subject to change and the methods that will be used to implement the modifications;
  - Performance evaluation protocols, to describe the processes that will be used to verify and validate the modified AI-DSF; Update procedures, to describe how the modifications will be implemented, either automatically, manually, or a combination of both, and the plan of communications to inform users
3. Impact assessment, which describes how the evaluation of benefits and risks, and the mitigation strategies associated with modifications are assessed, along with how they will be verified and validated through the activities detailed in the modification protocol. It should address how each modification impacts the device, including the risks of harm and bias; how each modification impacts others; and describe the cumulative impact of the implementation of all modifications.

As it will be detailed in section “Guiding Principles for PCCPs,” one of the guiding principles for PCCPs is stakeholder collaboration. Manufacturers, regulators, and end-users each have a determinant role in PCCPs. The developers are the ones responsible for the design, implementation, and documentation of PCCPs, and they should do it in line with guidelines published by regulators. The predefined modification should comply with the regulatory requirements and be transparent to end-users and regulators, providing them with evidence that the updates do not compromise safety or effectiveness. Engaging regulators in the early phases of the development of PCCPs may boost the possibilities of market approval, as they are the ones that define the criteria and procedures of acceptance and therefore can provide assistance for successful PCCPs. Finally, the end-users also play a role since they are the ones who benefit from PCCPs and can attest to the safety and effectiveness of the modifications implemented in the PCCP. Their role can be particularly relevant in postmarket surveillance mechanisms, considering they can be the first ones to identify problems and trigger potential modifications.

With PCCPs, manufacturers must plan the expected modifications and updates to AI-ML software, reducing some of the uncertainty intrinsic to such technologies. This brings benefits for the end-users, who are aware of the potential updates of a device and reduces uncertainty for developers and regulators, creating a clearer pathway for implementing improvements. Other benefits of PCCPs are their structured nature and the transparency they provide to all stakeholders, which increases trust and confidence between them. If a device has a PCCP, the stakeholders are aware of the possible updates and modifications and the corresponding monitoring and validation plans that will ensure the safety and effectiveness of the device.

**Figure 3.**
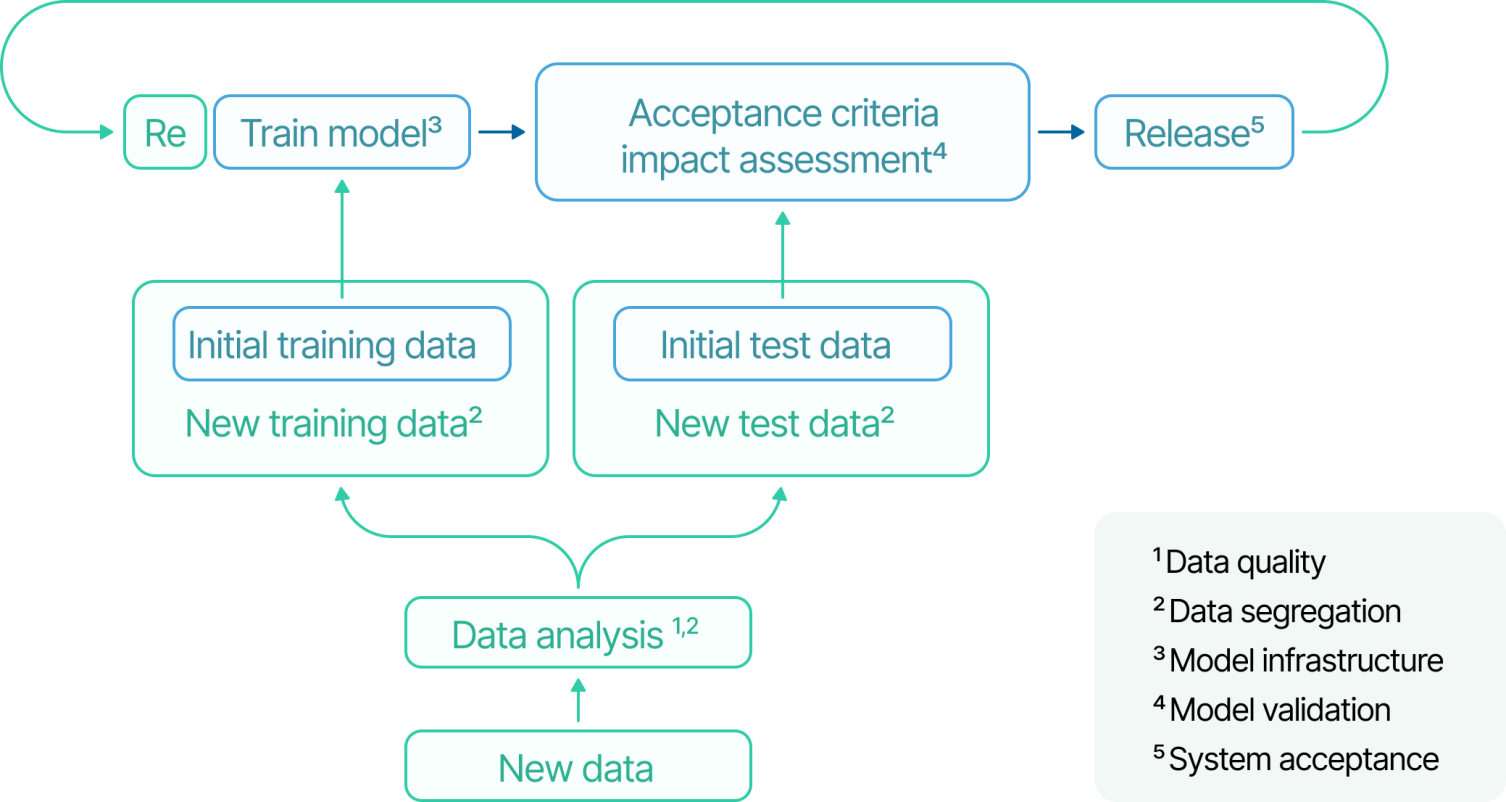
Locked model (blue) and continuous learning model (green).

By embedding PCCPs as a crucial tool within regulatory frameworks, manufacturers can reduce inefficiencies associated with frequent resubmissions while maintaining robust oversight, established mainly through transparency of intended refinements among regulators, manufacturers, and end-users, fostering trust in AI-driven health care innovations. A locked model is trained with the initial training data, it is tested, and if the acceptance criteria are met, the model is validated and released after approval. If new data are gathered or any other modification is implemented in one of the 5 stages, manufacturers would require a new market submission. With a PCCP, it is possible, for example, to update the model without the need for further market submissions. The new data are analyzed to ensure they have the quality required and respect the defined criteria. Then the data have to be segregated, and afterward, the model can be retrained. A new test set is created with previous and new data, which is used to ensure that the model matches the acceptance criteria defined in the modification protocol section, and if so, the model is released without further regulatory approval [16] (Figure 3).

## Guiding Principles for PCCPs

### Principle 1: Transparency and Explainability

Manufacturers and developers must provide regulators and end-users with detailed information regarding the components of the AI-DSF that are prone to changes and the corresponding impact. For the approval of a market submission with a PCCP, it is essential to provide clear and appropriate information, along with detailed plans, to users and other stakeholders to ensure that they are aware of the device’s performance and intended use before and after changes are implemented [5]. Regulatory expectations emphasize the necessity of version control and traceability to maintain oversight of modifications and ensure accountability. Proper documentation of each change is required to track updates over time, facilitating regulatory compliance and enabling thorough postmarket monitoring. The description of PCCPs should be publicly available on device summaries, for example, on the PMA summary of the safety and effectiveness document, or the 510(k) summary, or on the De Novo decision summary, depending on the pathway of premarket authorization. This description should include sufficient detail to provide transparency to stakeholders regarding safety and effectiveness. FDA recommends the inclusion in the public-facing documents of a summary with the following information: planned modifications, testing methods, performance requirements for the implementation of the modification, and means to communicate changes implemented following the authorized PCCP [6].

It is recommended that the labeling section of the market submission has a statement to inform that the device includes AI-ML technologies and has an authorized PCCP. In this manner, users are aware of the possibility of software updates and modifications that impact the device’s performance, inputs, or use. FDA also recommends the inclusion of the following information in the labeling section to ensure transparency [6]:

- A description of the modifications made, including the rationale, supporting evidence, data used, changes in inputs and or outputs, updated performance, and validation methods;
- A version control framework that ensures all changes are documented systematically, including timestamps, revision history, and regulatory justifications;
- An overview of the process followed to implement the modifications;
- An explanation of how the implemented modification will be communicated, including updated instructions for use and version history tracking.

Beyond documentation, manufacturers should implement governance mechanisms to ensure that version control processes are both proactive and auditable. This involves establishing dedicated teams responsible for monitoring modifications, conducting impact assessments, and maintaining alignment with evolving regulatory requirements. In addition, AI-ML model updates should be linked to a structured approval pipeline, ensuring that any modification—whether minor parameter adjustments or major retraining efforts—is systematically reviewed and justified before deployment [17]. These efforts help mitigate risks associated with unintended model drift, bias accumulation, or performance degradation, reinforcing the safety and reliability of AI-driven solutions.

### Principle 2: Risk Management

Continuous learning models require structured risk assessment frameworks to address concerns such as data drift and algorithmic bias. To increase the reliability and value of a PCCP, its design and implementation should be driven by a risk-based approach with proper principles of risk management. Performing a risk re-evaluation of the device after modifications is essential to determine the potential impact on the overall system’s safety, effectiveness, and compliance with regulatory standards.

PCCPs must align risk categorization with regulatory classifications and include a structured risk management process that is reflected in the impact assessment section. By providing a comprehensive analysis, manufacturers can demonstrate that the proposed modifications will not introduce new, unmitigated risks, thereby ensuring compliance with regulatory standards and maintaining device safety [6].

The International Medical Device Regulators Forum uses a risk categorization principle based on 2 key factors: the significance of the information provided by the medical device to the health care decision and the state of health situation or condition. The former identifies the intended use: to treat or diagnose, drive clinical management, or inform clinical management. The latter identifies the intended user, disease, and population: critical, serious, or non-serious. Using these factors, the SaMD is classified into four categories, ranging from lowest (I) to highest risk (IV), as shown in Table 2 [3].

**Table 2.** Software as a medical device risk categorization based on significance of information and health situation or condition.

State of health care situation or condition	Significance of information provided by SaMD^a^ to health care decision		
	Treat or diagnose	Drive clinical management	Inform clinical management
Critical	IV	III	II
Serious	III	II	I
Nonserious	II	I	I

a SaMD: software as a medical device.

In 510(k) premarket submission, the risk of the planned changes should be categorized similarly to the one used for the predicate device, and this risk category should not change. For example, it would not be feasible to include PCCP modifications that increase the risk category of the device. By providing a comprehensive analysis, manufacturers can demonstrate that the proposed modifications will not introduce new, unmitigated risks, thereby ensuring compliance with regulatory standards and maintaining device safety [6].

### Principle 3: Robust Change Protocols

Modifications can be either manual, automated, or a combination of both (hybrid). Despite its type, it is important to define a robust protocol to define when and how to ensure the implementation of modifications. The modification protocol section of PCCPs should include this information on the retraining practice’s part, where, among other information, the manufacturers should specify the triggers for retraining. Manufacturers should define clear triggers for updates, ensuring that changes are evidence-based and justified. Some examples of recognized triggers can be the acquisition of a given amount of new data, detection of drift in data, significant performance deviations (such as metrics degradation), or even a fixed cadence of weekly or monthly. In addition, modifications should consider the impact of new regulatory requirements, emerging best practices, or advancements in technology. This approach ensures that modifications remain effective and aligned with the device’s intended use and regulatory requirements while maintaining the safety and efficacy of the AI-enabled software functions [5,6]. .

### Principle 4: Stakeholder Collaboration

In 2024, the FDA published a guidance called “Requests for Feedback and Meetings for Medical Device Submissions: The Q-Submission Program” to outline the mechanisms available to manufacturers to get feedback for medical device premarket submission [18]. This guidance encourages manufacturers to leverage the introduced Q-Submission Program to get feedback on a proposed PCCP before submitting a marketing submission. In this way, regulators are engaged early in the development cycles, which can facilitate the approval processes for PCCP and allow manufacturers to proactively address any concerns that may arise during the development of their plans. The Q-Submission Program can be used to tackle issues and doubts that may arise in the development of a PCCP. Topics such as proposed changes to the PCCP, considerations for automatic updates, and modifications to the intentions of use are some examples of how appropriate FDA review division members can help manufacturers through the Q-Submission Program [6]. Early engagement with regulatory bodies and open communication between technical and regulatory teams play a critical role in expediting the approval of PCCPs. In this way, regulators are engaged early in the development cycles, which can facilitate the approval processes for PCCP and allow manufacturers to proactively address any concerns that may arise during the development of their plans. To ease and foster collaboration between stakeholders, the FDA intends to harmonize information and lay a foundation for PCCPs to support innovations in the digital health space [5].

Considering opinions and inputs from all stakeholders, the quality and integrity of PCCPs will improve, and international harmonization and stakeholder consensus on the core concepts of PCCPs will help support the advancement of responsible innovations in the digital health space. This iterative review process between stakeholders fosters regulatory confidence in the manufacturer’s approach, enabling a smoother transition from development to market authorization, whilst assuring clarity on what constitutes a “significant” versus “minor” modification under a PCCP, reducing uncertainty regarding future update approvals. Significant changes, contrary to minor ones, include modifications that could significantly affect the safety or effectiveness of the device, such as changing the device’s intended use. These changes often require a new marketing submission, which highlights the importance of early engagement with regulatory entities to verify the feasibility of using PCCPs. By promoting collaboration among stakeholders, the FDA supports the advancement of responsible innovations in the digital health industry, ensuring that new technologies meet regulatory standards while addressing patient needs [5].

### Principle 5: Continuous Monitoring and Feedback

Developing postimplementation monitoring systems and integrating real-world evidence are key aspects to consider before the market submission of a medical device with a PCCP. Manufacturers must develop comprehensive postmarket surveillance plans and procedures detailing real-world monitoring strategies and notification requirements for malfunctions and other safety concerns. It is the responsibility of the manufacturer to ensure ongoing safety and effectiveness of the device, which can be accomplished by implementing monitoring plans for adverse events, as well as performance metrics tracking. These monitoring activities highly depend on the type of device and the type of updates (whether manual or automated, and whether they are applied globally or locally) [6].

In addition, the manufacturers should also ensure that the modifications are implemented according to the PCCP, which highlights the need for well-defined monitoring means to guarantee compliance with the requirements and specifications set in the PCCP.

After approval of a PCCP, it is recommended that manufacturers develop standard operating procedures (SOPs), where they set the monitoring and evaluation processes. These SOPs should specify methods for collecting data on device performance, safety, and efficacy, as well as the management of this data. In addition, SOPs should also identify the personnel in charge of monitoring activities and the corresponding roles and responsibilities. With the implementation of these procedures, manufacturers guarantee that the modifications are in line with the PCCP and that the device consistently meets established safety and performance standards without requiring reapproval [2]. In the update procedures included in the modification protocol, the device monitoring plan should be detailed, and manufacturers should [6]:

- Detail the methods to track the events triggered by PCCP modifications, to ensure timely identification of issues;
- Have a risk-based plan to monitor the real-world performance of the device, allowing for adaptive responses to emerging patterns of data;
- Specify how changes in safety and effectiveness will be monitored and how frequently, including the frequency of these assessments;
- Detail the steps to tackle unexpected performance deficiencies or safety hazards, ensuring proactive risk management;
- Define criteria and plans to reverse an update and reset devices to previous versions, if needed, to maintain device integrity and user confidence.

Postmarket surveillance and monitoring can benefit from feedback provided by end-users, as it often offers valuable insights into device performance and potential issues. However, manufacturers must also implement strategies that allow them to monitor devices independently of user input. One effective approach is automated logging, in which devices securely transmit operational and performance data directly to the manufacturer’s systems. At the same time, manufacturers should facilitate the contribution from end-users with simple mechanisms for flagging issues. Integrating these reports with automatic device logs and relevant data can ease the process for both the manufacturer and the user, ensuring that any problems are captured quickly and with sufficient context for effective resolution. By prioritizing continuous monitoring and feedback mechanisms, manufacturers can sustain the safety and efficacy of their devices, engage effectively with regulatory agents, and support ongoing innovation within the medical device field.

## Implications of PCCPs in AI-ML Development

As mentioned before, transparency is one of the principles for PCCPs. Clear labeling is essential to provide information to users, including details describing the data used, data sources, the input variables and their relevance, the rationale behind model decisions, the aim of the output, and the supporting performance evidence. PCCPs require that manufacturers explicitly outline possible modifications, describing how they are going to be implemented and assessing their impact on performance, safety, and effectiveness. Without this, regulators will not give market approval. In this way, PCCP ensures enhanced trust, which builds confidence among users and stakeholders who have more access to details of the AI-ML technology that would be undisclosed if a PCCP were not submitted in the market approval submission [19].

During the development of PCCPs, manufacturers should accurately define the intended use populations and provide demographic information (race, ethnicity, disease severity, gender, age, etc), and intended environments of use (eg, hospitals, clinics, telemedicine, laboratories, radiology, and ICUs). With this approach, it is guaranteed that the device continues to operate on the defined and planned populations and environments even as modifications occur. To mitigate potential bias, it is required that manufacturers present details in PCCPs showing that the algorithms were tested on representative populations. The submission of a PCCP for market approval ensures that the device remains safe with the planned modifications, which is critical for addressing bias and ensuring the fairness of AI-ML technologies [19].

Overall, PCCPs ensure that the device will remain safe and performant under the specified modifications and enable manufacturers to use continuous learning AI-ML technologies. Thus, by integrating structured modification protocols, PCCPs foster responsible AI innovation while ensuring that safety and efficacy are not compromised.

Another benefit of PCCPs is the possibility of reducing delays and expediting times for approvals by minimizing the number of submissions for market approval. When the manufacturers submit a PCCP, it ensures that the AI-DSF will not need reapproval after updating the device with detailed modifications. In this manner, PCCPs have the power to streamline regulatory processes, reducing the workload of regulatory entities and, consequently, decreasing the waiting times for approval.

**Figure 4.**
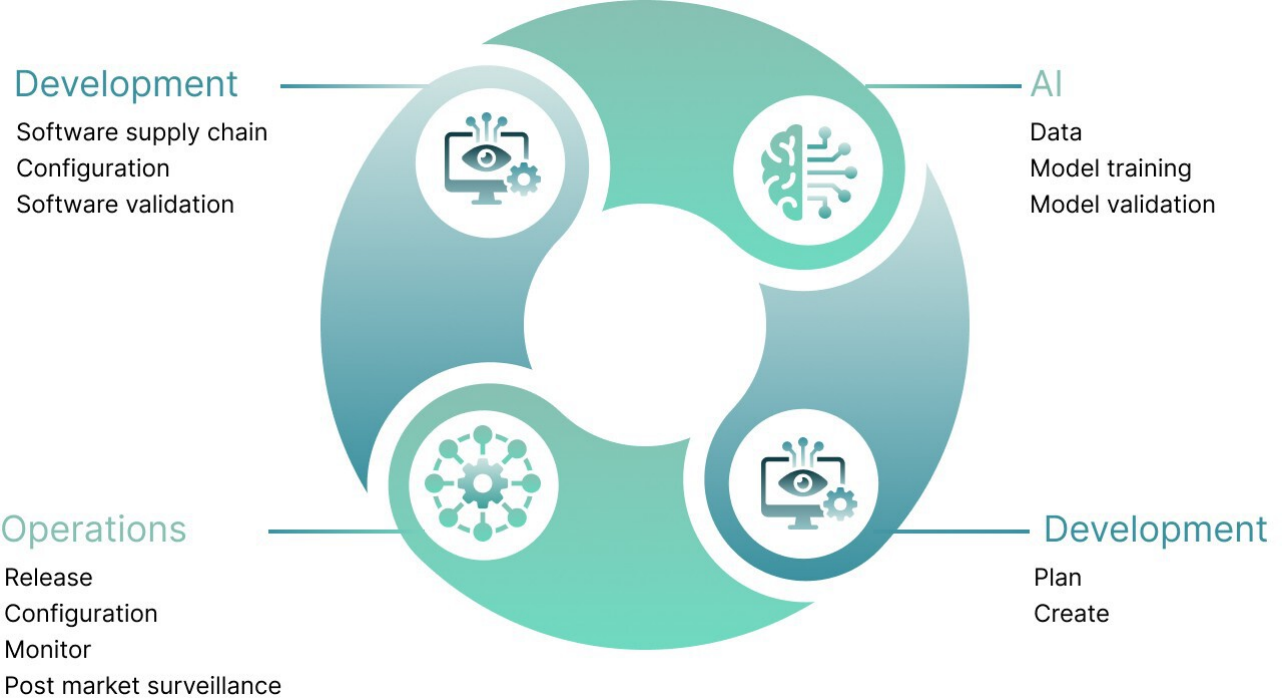
Continuous integration and delivery in machine learning.

To get the most out of AI-ML technologies, it is important to integrate PCCPs into modern software development practices such as continuous integration and delivery (CI-CD), which is a development practice where code changes are automatically built, tested, and deployed. It enables fast, frequent, and reliable updates and releases. CI-CD facilitates the validation of AI-DSF with automated testing, version control, validation gates, monitoring, and feedback loops. Thus, manufacturers of SaMD need to shift from waterfall-style development to more agile approaches to remain competitive and fully harvest the benefits of AI-ML technologies. The application of CI-CD in ML is already well established under a continuous workflow (Figure 4) [20].

However, the implementation of CI-CD with regulatory compliance is challenging, particularly in medical software. To facilitate regulatory compliance, manufacturers should:

- Design a model architecture that facilitates modifications whilst maintaining compliance;
- Enforce procedures and generate automatic evidence, such as required documentation, reports, and audit trails to ensure that modifications are automatically documented and that they are in compliance with regulations;
- Implement risk analysis in the research and development process, including risk identification, evaluation, and development of mitigation plans;
- Automate documentation and testing processes to streamline compliance efforts;
- Keep traceability across and within different phases (design, training, validation, deployment, and postmarket monitoring).

To tackle some of these challenges, it is possible to use DevOps or MLOps-based deployment systems, which can improve automation and traceability [21].

This integration of PCCPs and CI-CD allows for faster time-to-market, enhanced quality, and increased flexibility [22]. This synergy positions AI-ML technologies as both agile and accountable, enabling responsible innovation in digital health while maintaining public trust and regulatory confidence.

## Case Studies and Applications

The theoretical framework and guiding principles PCCPs provide a clear foundation, show their importance, and their true value. However, the benefits, limitations, and challenges can be better understood with examples of use in real-world scenarios. In this way, it is possible to better understand how they are implemented under varying conditions and how they adapt to specific contexts.

The FDA already approved several AI-DSFs for the health care sector, including tools to enhance diagnostic accuracy, automate processes, predict patient outcomes, improve workflow efficiency, and deliver personalized treatment plans.

For example, Clarius Mobile Health, a company specializing in Medical Equipment Manufacturing, develops products to make ultrasound imaging accessible, efficient, and convenient for health care professionals. Recently, they received FDA approval for Clarius OB AI, an AI tool that performs fetal biometry measurements to estimate fetal age, weight, and growth intervals. It can be used with the company’s Clarius C3 HD3 wireless handheld ultrasound scanner and allows obstetrical prenatal monitoring.

The 510(k) summary submitted by the company clearly describes the device, the indications for use, and a summary of the PCCP. It includes 4 modifications and the corresponding rationale, testing methods, and impact assessment, 3 of which were intended to improve performance: modification of model architecture, modification of model training methods and parameters, and modification of postprocessing algorithms. The company is going to assess this with a comparison of the model and clinical performance metrics between the original AI model and the modified one in clinical testing. For these modifications, a benefit-risk analysis was performed, identifying improved performance and generalization as benefits and overfitting and unintended bias as risks. For these risks, they also established a mitigation plan, including regularization techniques, cross-validation, dropout to avoid overfitting, and internal testing and verification to mitigate biases. The fourth modification is related to data input sources, and it intends to enable the use of the Clarius OB AI on updated versions of the 510(k)-cleared Clarius Ultrasound Scanner system. The modification protocol includes retraining of the model with new data sources and internal and clinical testing. In the impact assessment, the risk-benefit analysis is outlined, mentioning enhanced compatibility and flexibility as advantages, and data skewing and concept drift as risks, which will be mitigated with internal testing and verification datasets within the intended patient population.

Finally, the company also clarifies how they will ensure transparency and keep users informed by communicating modifications via the Clarius App (Clarius Mobile Health Corp) software update notifications and through updated labeling [23].

Another example is the model from Beacon Biosignals, a health technology company focused on accelerating the development of therapies for neurological, psychiatric, and sleep disorders. They have received FDA 510(k) clearance for SleepStageML, an ML software that automatically stages sleep from electroencephalogram (EEG) signals of clinical recordings to aid in the diagnosis and evaluation of sleep and sleep-related disorders. The software included an authorized PCCP comprising the following modifications, all of them to improve sleep staging performance within the intended use population [24]:

- Update of ML model: achieved with retraining with new training data, with new hyperparameters, loss functions, and optimizers, and with different model architectures;
- Update signal processing steps: accomplished with updates to the digital signal processing steps applied to the EEG signal before being used by the ML model;
- Update probability postprocessing: implemented with modification of the methods used to generate sleep stages from the model output;
- Update of signal quality check: achieved with the update of the thresholds used to check that the input EEG signals are analyzable

The PCCP also identified which of these modifications trigger the retraining of the model and states that the tests of modification will include software verification and validation testing, where clinical performance will be assessed. The company clearly defined the acceptance criteria for each of the modifications and clarified how updates would be communicated to users, ensuring that guiding principles 1, 3, and 5 were fulfilled.

## Emerging Fields

The use of AI-ML technologies is not confined to health care. While health care remains the most mature and regulated domain for PCCP implementation, illustrated by concrete FDA-cleared examples such as Clarius OB AI and Beacon Biosignals’ SleepStageML, the underlying principles of structured, prespecified change control are not unique to health care.

As AI-ML permeates diverse sectors, the need for a structured yet flexible approach to managing updates and evolutions becomes universal. Adopting PCCPs beyond health care could streamline innovation, reduce regulatory bottlenecks and complexity for manufacturers, improve time-to-market, and ensure trust in AI systems that operate in dynamic, real-world environments.

Although the regulatory context and risk thresholds might differ, the conceptual rationale for PCCPs can be meaningfully extended to other domains that rely on systems that must adapt safely to changing conditions and face similar challenges. Whether in finance, transportation, or education, the underlying question is the same: how can innovation continue without compromising safety and trust.

A comparative overview of how PCCPs may be applied across sectors, highlighting concrete examples of use cases, potential benefits, associated risks, and the current state of regulatory oversight, is provided in Multimedia Appendix 1. This cross-sectoral perspective underscores the generalizability of PCCPs as a framework for trustworthy AI, while reinforcing the unique depth of implementation achieved to date in biomedical applications.

## Challenges and Limitations

Identifying, monitoring, and managing changes to ensure the protection of all stakeholders is the primary regulatory challenge of AI technologies in the health care domain. Each stakeholder, regulators, manufacturers, health care providers, and patients values and focuses on different aspects and components, leading to misalignment between technical, ethical, and legal expectations. Therefore, it is essential in the regulatory ecosystem to involve regulators, professional organizations, and service providers [10]. Current regulations are usually country-specific, reducing the global applicability of AI-ML technology and potentially increasing inequalities, as some countries may benefit more than others from such technologies. Creating harmonized and global regulations and guidelines for modifications of AI-DSF will be a challenge in the future that will need the collaboration of stakeholders and governments worldwide.

The development of a PCCP is time-consuming and resource-intensive, demanding expertise across multiple domains: regulatory, technical, clinical, and quality assurance. Implementation costs extend beyond development. Continuous monitoring, verification, and validation of AI-ML updates under a PCCP demand ongoing investment in infrastructure (eg, data management systems) and personnel training. Real-world performance tracking—a core PCCP requirement—may necessitate additional software tools or third-party services, further elevating expenses. Manufacturers must also update quality management systems to align with PCCP processes, adding to operational costs. In addition, hospitals and other infrastructures also need to be updated since they handle their systems manually, providing data through informal reports rather than using automated or integrated systems. This increases upfront effort and costs for manufacturers, who must invest in establishing the necessary frameworks, tools, and documentation, imposing a high initial cost barrier. Furthermore, given the complex structure and requirements for the implementation of PCCPs, and the heterogeneous nature of AI-ML-enabled products and manufacturer operations, such costs are difficult to estimate beforehand. These expenses can strain small- to medium-sized enterprises, potentially limiting adoption to larger firms with greater financial capacity. While PCCPs aim to reduce long-term regulatory burdens by minimizing resubmissions, the initial and sustained financial outlay poses a challenge, particularly without clear cost-benefit data due to PCCPs’ recent emergence. Regulatory bodies like the FDA acknowledge potential cost savings (eg, fewer submissions), but without peer-reviewed studies quantifying these against implementation expenses, the economic feasibility remains speculative. This level of professionalization associated with PCCP implementation may be manageable for large corporations with dedicated regulatory departments but represents a substantial hurdle for startups and small and medium enterprises (SMEs) seeking to enter the market. Consequently, the financial burden of PCCP adoption risks consolidating innovation within a limited pool of well-capitalized firms, potentially stifling broader market diversity and competitiveness.

In addition, PCCPs are complex to implement since AI-ML technologies have transboundary elements and, therefore, must comply with different regulations and jurisdictions. It is difficult to manage transnational cooperation and jurisdictional sovereignty [10]. For example, it is easier to get market approval in the EU than in the United States, which motivates companies to launch their product outside the United States. The FDA approval is often a longer and more expensive process, and if the product is intended to be used in multiple markets, manufacturers face additional challenges since separate regulatory approvals may be required in each country [25,25].

Furthermore, the lack of global harmonization in regulatory frameworks further complicates the implementation of PCCPs across jurisdictions. The United States and the European Union, while both moving toward adaptive regulatory approaches, diverge significantly in terms of approval processes, expectations for modification protocols, and the maturity of their PCCP-related guidance. The FDA’s framework emphasizes premarket clarity with detailed requirements for modification protocols, while the EU regulatory landscape—shaped by the MDR and evolving through the AI Act—still lacks equivalent specificity for AI-DSFs.

These disparities can force manufacturers to create jurisdiction-specific versions of their PCCPs or maintain parallel documentation and risk assessment pipelines, increasing operational overhead and regulatory uncertainty. Moving forward, mutual recognition mechanisms or modular PCCP templates adaptable to both United States and EU standards could help reduce duplication and facilitate safer, faster cross-border deployment of AI-ML-enabled medical technologies.

Moreover, these technologies are constantly changing and evolving rapidly, which urges the need for adaptable regulations that consider such changes. The new regulatory approaches should complement existing protocols, providing mechanisms to monitor changes and fill the gaps in the current governance regulations. New regulations should be flexible and accommodate rapid changes, therefore supporting innovation but without adding burden to pre-existing and well-established frameworks.

## Future Directions

Approval of devices and products is associated with high waiting times for AI-DSF, and in the health care field, these waiting times tend to be even higher. For example, the average median wait time between the date the FDA receives the complete application and the decision date is 125 days [26]. However, the process to get approval begins much before the submission of the complete application and is often iterative. This highlights the need for faster mechanisms to approve market approval submissions for AI-ML technologies since such waiting times are sufficient to hinder the applicability, benefits, and use of such technologies. In addition, considering the adaptive nature of AI, it is not feasible to submit a market approval for each modification on the device.

Using a firm-based approach to regulate SaMD with AI-ML technologies shifts the focus of the regulation to the development process, giving more importance to quality systems and development processes. This seems to be the path to be followed by regulators for AI medical devices, enabling updates to existing approved products without the need for further approvals [27]. In addition, PCCPs are an interesting solution to avoid the need for market approval submission for each modification while maintaining the safety and effectiveness of the device. However, regulatory entities have to decrease waiting times for approval and make such a process more efficient. Using AI to manage PCCPs and decrease waiting times could be a tool to consider. A possible alternative or complement is to transfer the process of some devices to third parties, possibly automatically with AI, which would allow the FDA to focus on higher-risk devices, maintaining high-quality reviews and appropriate timing [28]. Establishing a framework for third-party certification of SaMD with lower risk would assist the FDA in tackling problems of internal expertise, streamlining the certification process, and improving the level of expert guidance the agency can offer [27]. This solution also introduces its own risks, such as conflicts of interest. Thus, it is important to request accreditation of certifiers, periodic audits, and publicly disclose the evaluation methodologies, criteria, and decision processes.

In addition, considering that the performance and functionality of AI-ML technologies highly depend on reliable and good data, it could be of great interest that jurisdictions create an entity responsible for facilitating the creation of datasets for AI development that adhere to regulations and standards. This would ensure that data comply with regulatory requirements for the development of AI-ML technologies and, consequently, reduce the premarket scrutiny of such technologies, considering that the models were developed using reliable datasets [27]. In the EU, there are some projects that aim to accomplish this. Some examples are CHAIMELEON [29], ProCancer-I [30], and EUCAIM [31], which can foster collaborative research, drive innovation, and ease the premarket examination.

As mentioned in the previous section, an issue with the mentioned regulations is that they are only valid for a given country, reducing the applicability of the AI-DSF and increasing inequalities. Thus, it is essential to develop regulations that comply with and integrate global standards. Governments should state which authorities are responsible for regulation and which components of AI systems are subject to it. Governing bodies should also clarify which existing regulatory mechanisms must or could be applied to systems with AI-ML technologies. For example, regulators can clarify how medical device regulation can be applied to AI-ML systems or add recommendations to existing approval requirements to monitor modifications of AI-ML-enabled devices, which was accomplished by FDA draft guidance [6]. More regulations and guidelines are required to help manufacturers develop PCCP for AI-ML technologies in a way that they integrate and comply with global standards, mitigating inherent risks of these technologies and ensuring that these tools are reliable, safe, and used worldwide. The design of new regulatory frameworks for AI-ML SaMD should not only involve medical device software experts but also experts on postmarket surveillance, real-world performance measurement, and clinical evaluation, as well as patient representatives.

In the future, it is also relevant that jurisdictions develop novel validation strategies that enable faster decisions on whether or not to approve an AI-DSF while maintaining safety and quality. Postmarket surveillance remains a key point in AI-ML technologies, where it is essential to guarantee that the devices AI-DSF are performing up to the quality standards to which they were approved and that updates do not decrease safety or effectiveness.

Creating platforms or templates to help manufacturers and companies create PCCPs compliant with FDA guidelines and regulations can also be an opportunity to foster innovation and the use and approval of AI-DSF, particularly in health care. Providing instructions and procedures to complete the 3 PCCP sections (Description of modifications, modification protocol, and impact assessment) and finding a way that manufacturers can easily replace text with their product-specific information can be a valuable tool to assist the market approval of AI-DSF with planned modifications. There is already an application for the lifecycle management of software called Ketryx (Ketryx Corporation) that helps companies build FDA-regulated software. The creation of more platforms and tools to automate and facilitate manufacturers and regulators to deal with changes to AI-ML technology can be of great interest to foster innovation. In addition, the creation of such templates, tools, and platforms can help SMEs overcome the limitations mentioned in the previous chapter by providing them with more support to adopt PCCPs. Regulatory entities should also foster the creation of consortia and resources to share knowledge and for mentoring.

In the future, it would be important to conduct studies to report and analyze the costs of implementing PCCPs and compare them with the traditional paths. This can help manufacturers, particularly SMEs, identify the investment needed and understand if submitting repetitive market submissions requires a higher investment than implementing PCCPs.

There are many regulations, including rules from public authorities and the private sector. Generally, AI-related regulations should be formulated by public bodies and regulatory oversight should adapt to the advancements of technology to avoid slowing down innovation. Future regulations should also take into account the domain of generative AI, which may need distinct legislation and guidelines. Current regulations and PCCPs are mainly focused on AI-ML models and do not address generative AI. Considering the fast adoption of such technology, it is essential to create standardized regulations to tackle problems of reproducibility, transparency, data privacy, security, and intellectual property. In fact, the FDA released an executive summary with a focus on the oversight of generative AI-enabled devices, which shows the pertinence of the topic and the need for regulations and guidelines [32]. Despite the current limitations, PCCPs could be adapted by requiring more granular change protocols that explicitly define permissible updates to model architecture, prompt-handling mechanisms, and training datasets. Furthermore, the principle of continuous learning of PCCPs is essential for generative AI-enabled devices, as robust postmarket performance monitoring can ensure safety and effectiveness after market entry. Monitoring mechanisms could be extended to include output sampling and quality benchmarking over time, ensuring that generative components maintain compliance and reliability.

## Concluding Remarks

Considering the continuous and adaptive nature of AI-ML technologies, PCCPs emerge as an essential tool in the development and regulation of AI-DSF, ensuring that the benefits of continuous learning are exploited and systems evolve efficiently and safely. It empowers manufacturers to update AI-DSF within the constraints of safety and effectiveness without the need to make additional premarket submissions. This framework reduces the time and resources needed by manufacturers and regulators to approve AI-DSF.

Moreover, PCCPs enhance transparency, compliance, and trust among stakeholders and reduce regulatory burden. Promoting a culture of transparency and collaboration among all stakeholders, including developers, users, and regulators, can enable the creation of regulations, which are essential to ensure that AI-ML technologies are developed and used responsibly, with ensured safety, effectiveness, and reliability.

Regulators should actively seek input from stakeholders to gain insights into the challenges associated with developing, deploying, and monitoring AI-ML technologies. In addition, governments should be encouraged to disseminate best practices and standards for the use of AI and foster a research ecosystem that leverages public-private partnerships [25]. Acknowledging that adherence to regulatory guidelines can be time and resource-consuming, regulations must remain flexible to promote innovation and are developed globally.

With the current evolution of the adoption of AI-ML technologies, manufacturers with PCCPs and CI-CD pipelines will be well-positioned to deliver superior products. The future of AI-ML SaMD will be shaped by automation, adherence to regulations, and continuous evolution for the benefit of the patients and health care providers.

## Supplementary material

10.2196/76854 Multimedia Appendix 1 Application of predetermined change control plans in emerging fields.

## Abbreviations

AI: artificial intelligence
AI-DSFs: AI-enabled device software functions
CI-CD: continuous integration and delivery
EEG: electroencephalogram
EU: European Union
FDA: Food and Drug Administration
MDR: Medical Device Regulation
ML: machine learning
PCCP: predetermined change control plan
PMA: premarket approval
SaMD: software as a medical device
SME: small and medium enterprises
SOP: standard operating procedure
