# Supplementary material for: Predetermined Change Control Plans: Guiding Principles for Advancing Safe, Effective, and High-Quality AI-ML Technologies
Source: JMIR AI. 2025 Oct 31;4:e76854. doi: 10.2196/76854 (PMC12577744; doi:10.2196/76854)
Supplement: Multimedia Appendix 1 [file ai-v4-e76854-s001.docx]

| **Industry** | **Use Cases** | **Potential Benefits of PCCPs** | **Industry** | **Use Cases** |
| --- | --- | --- | --- | --- |
| Financial Services | Fraud detection and prevention and algorithmic trading [1] | For the establishment and approval of fraud detection mechanisms, PCCPs can redefine what constitutes a modification, and guarantee that development and validation requirements are met. Its emphasis on version control further enables tracking changes and rolling back to previous versions when necessary, demonstrating particular value in domains such as fraud detection, where frequent updates are required to counter fraud drift and evolving threats [2,5]. | Highly dynamic nature of financial markets poses risk of model drift, overfitting, and unintended bias when updating AI/ML systems. Strong boundaries and continues monitoring are required. | There is no AI-specific regulation; instead, oversight relies largely on existing frameworks covering prudent business practices, model risk management, investor and consumer protection, and data privacy [2]. Within the EU, this oversight is complemented by the prescriptive  rules-based approach of the AI Act, while in the US, financial authorities such as the United States Securities and Exchange Commission have adopted a more principles-based stance, exemplified by the proposed rule on predictive analytics [3,4]. Beyond these major jurisdictions, the Organisation for Economic Co-operation and Development has emphasized the importance of reviewing and reinforcing current frameworks rather than introducing entirely new regulatory systems [2]. |
| Digital Health and Wellness | Fitness trackers, dietary planners, Cognitive Behavioral Therapy interventions based on user-generated data (e.g., activity levels, sleep patterns, journal entries, wearable metrics) [6–8]. | PCCPs could formalize how these apps evolve adjustments within its therapeutic context specify acceptable changes (e.g., algorithm adjustments within a therapeutic range), testing methods (e.g., A/B testing with user cohorts or clinical validation), and risk assessments (e.g., avoiding prompts that could heighten anxiety); ensures apps remain effective and safe, fostering trust among users, vendors and regulators. | The definition of "safety" is subjective in non-medical devices, compared to medical devices, requiring regulators to set clear thresholds, possibly under consumer protection or digital health guidelines. | Unlike medical devices, regulated under strict frameworks like FDA’s, these apps often fall into a regulatory gray area [7], yet their influence on user behavior and well-being is significant, particularly in mental health contexts like stress management or therapy support [8]. |
| Automotive | Self-driving vehicles, real-time navigation, perception (e.g., pedestrian detection), decision-making (e.g., lane-changing logic), and adaptive responses to road and environmental conditions [9,10]. | Manufacturers could refine AI models as new driving scenarios emerge (e.g., construction zones), maintaining safety without halting deployment. Regulators would gain visibility into changes, ensuring compliance with standards like ISO 26262 (Road Vehicles - Functional Safety) [10]. | In this area, AI errors could lead to damages, injuries and fatalities, so strong boundaries and continuous monitoring are required [9,10]. | Current automotive regulations, such as those from the US National Highway Traffic Safety Administration [9], focus on premarket certification, but AI-driven vehicles require continuous updates post-deployment. PCCPs would need rigorous boundaries (e.g., no changes to core braking logic without new approval) and robust postmarket surveillance integrated with performance monitoring and incident reporting. |
| Climate and Environmental Services | Climate Modeling and Environmental Monitoring | PCCPs could be used to ensure reliability during updates, bridging the gap between scientific advancement and regulatory trust [13]; a structured approach would allow researchers to refine models swiftly without lengthy re-approvals, supporting timely climate action. | Robust cross-checking against historical climate trends and monitoring (e.g., tracking model accuracy post-update) are required to establish trustworthiness. | AI/ML outputs often inform high‑stakes policy decisions [12] and are governed by frameworks like the EU AI Act and national environmental data standards, which demand transparency, auditability, bias mitigation, and continuous monitoring. Thus, updates to climate models must include rigorous validation and documentation that meet both scientific rigor and regulatory compliance, enabling policy makers to rely on AI‑driven insights. |
| Utilities | Optimization of power distribution, demand prediction, and integration of renewable sources in the energy mix [11]. | PCCPs could enable pre-approved updates to forecasting and load-balancing algorithms, ensuring reliability while adapting to new data (e.g., weather patterns, consumer trends); it would establish acceptable changes (e.g., recalibrating load-balancing algorithms), testing methods (e.g., simulations of peak load scenarios to verify stability), risk management (e.g., preventing blackouts from over-optimistic forecasts), and monitoring (e.g., real-time grid performance metrics) [11]. | Regulatory lag complicates oversight, and maintaining grid stability during frequent AI-driven updates requires robust testing (e.g., peak load simulations) and continuous monitoring [11]. | Traditional energy regulations often lag behind rapid technological advances, necessitating flexible yet controlled frameworks. Bodies such as FERC and ENTSO‑E demand rigorous validation, transparency, and real‑time data sharing for AI‑driven grid updates. A dynamic governance model that integrates regulatory oversight with automated testing and continuous monitoring could balance innovation with grid security best practices and regulations. |
| Education | AI-driven tutoring system and personalize learning experiences [14] | PCCPs could ensure controlled updates to recommendation engines, maintaining fairness, alignment with curricula, and effectiveness of learning outcomes while enabling innovation [15]. | Updates must avoid introducing bias or inequity [14] and ensure that learning outcomes remain fair, effective, and aligned with educational standards [15]. They also carry risks to student privacy, demand algorithmic transparency for educators and parents, and require human oversight to prevent over-reliance on AI. | The education sector is governed by data privacy laws such as FERPA in the US and GDPR in the EU, alongside national education policies that mandate transparency and fairness for AI tools. These regulations require rigorous data governance, algorithmic accountability, continuous monitoring, and human oversight to protect student privacy and ensure equitable learning outcomes. |
| Supply Chain & Logistics | Predicting demand, optimizing shipment routes, managing inventory, adapting to disruptions like port closures or demand spikes [16]. | PCCP could ensure updates maintain reliability and compliance, preventing economic ripple effects [17]. PCCP for a logistics AI might predefine updates to routing algorithms (e.g., adapting to fuel cost changes) or demand forecasts (e.g., holiday surges). The plan would specify changes (e.g., optimizing delivery paths), validation methods (e.g., simulated delivery times), risk assessments (e.g., avoiding stockouts or delays), and monitoring (e.g., real-time tracking data). | The dynamic nature of supply chain AI introduces risks such as cascading failures from erroneous demand forecasts or routing decisions, potentially leading to stockouts or delays. | AI systems in supply chain and logistics are subject to a complex regulatory framework, including US DOT agencies (FMCSA, NHTSA, FAA) and international bodies such as IMO, IATA, and ICAO. Cross‑border data must comply with GDPR and US privacy laws, while NIST cybersecurity standards guide AI protection. These regulations require auditability, transparency, and human oversight to ensure safety, trade compliance, and environmental stewardship. |

## References

1. Azzutti A. Chapter 18: Artificial intelligence and machine learning in finance: Key concepts, applications, and regulatory considerations. 2024. [doi: [10.2139/ssrn.4851418](http://dx.doi.org/10.2139/ssrn.4851418)]
2. Regulatory Approaches to Artificial Intelligence in Finance. OECD. 2024. [doi: [10.1787/f1498c02-en](https://doi.org/10.1787/f1498c02-en)]
3. Request for information and comment on financial institutions’ use of artificial intelligence, including machine learning. United States Government. 2021. URL: <https://www.federalregister.gov/documents/2021/03/31/2021-06607/request-for-information-and-comment-on-financial-institutions-use-of-artificial-intelligence> [Accessed 2025-01-15]
4. Conflict of Interest Associated with the Use of Predictive Data Analytics by Broker-Dealers and Investment Advisers. US Securities and Exchange Commission. 2023. URL: <https://www.sec.gov/rules-regulations/2023/07/s7-12-23> [Accessed 2025-01-15]
5. Artificial Intelligence Model Risk Management. Monetary Authority of Singapore. 2024. URL: <https://www.mas.gov.sg/-/media/mas-media-library/publications/monographs-or-information-paper/imd/2024/information-paper-on-ai-risk-management-final.pdf> [Accessed 2024-12-28]
6. Torous J, Bucci S, Bell IH, Kessing LV, Faurholt-Jepsen M, Whelan P, et al. The growing field of digital psychiatry: current evidence and the future of apps, social media, chatbots, and virtual reality. World Psychiatry. 2021 Oct;20(3):318-35. PMID: 34505369. [doi: [10.1002/wps.20883](https://doi.org/10.1002/wps.20883)]
7. De Freitas J, Cohen IG. The health risks of generative AI-based wellness apps. Nature Medicine. 2024 2024/4/29;30 PMID - 38684859(5):1269-75. [doi: [10.1038/s41591-024-02943-6](http://dx.doi.org/10.1038/s41591-024-02943-6)]
8. Tavory T. Regulating AI in Mental Health: Ethics of Care Perspective. JMIR Ment Health. 2024 2024/9/19;11:e58493. [doi: [10.2196/58493](https://doi.org/10.2196/58493)]
9. A Framework for Automated Driving System Testable Cases and Scenarios. National Highway Traffic Safety Administration (NHTSA). 2018. URL: <https://www.nhtsa.gov/sites/nhtsa.gov/files/documents/13882-automateddrivingsystems_092618_v1a_tag.pdf> [Accessed 2025-01-15]
10. ISO 26262:2018 Road Vehicles-Functional Safety. ISO. 2018. URL: <https://www.iso.org/standard/68383.html> [Accessed 2025-01-15]
11. Entezari A, Aslani A, Zahedi R, Noorollahi Y. Artificial intelligence and machine learning in energy systems: A bibliographic perspective. Energy Strategy Reviews. 2023 2023/01/01/;45:101017. [doi: [10.1016/j.esr.2022.101017](https://doi.org/10.1016/j.esr.2022.101017)]
12. AI Act. European Commission. 2024. URL: <https://digital-strategy.ec.europa.eu/en/policies/regulatory-framework-ai> [Accessed 2024-12-28]
13. Rolnick D, Donti PL, Kaack LH, Kochanski K, Lacoste A, Sankaran K, et al. Tackling Climate Change with Machine Learning. ACM Comput Surv. 2022;55(2):Article 42. [[doi: 10.1145/3485128](https://doi.org/10.1145/3485128)]
14. Miao F, Holmes W. Artificial intelligence and education guidance for policymakers United Nations Educational, Scientific and Cultural Organization (UNESCO): Paris, France. 2021. URL: <https://unesdoc.unesco.org/ark:/48223/pf0000376709> [Accessed 2025-01-15]
15. Cardona MA, Rodríguez RJ, Ishmael K. Artificial Intelligence and the Future of Teaching and Learning: Insights and Recommendations. Washington, DC: U.S. Department of Education, Office of Educational Technology, 2023. URL: <https://www2.ed.gov/documents/ai-report/ai-report.pdf> [Accessed 2025-01-15]
16. Chopra S. Supply Chain Management: Strategy, Planning, and Operation. 7th edition. Pearson Education Limited; 2019. ISBN: 0134732510
17. Freight Analysis Framework. United States Department of Transportation (USDOT). URL: <https://ops.fhwa.dot.gov/freight/freight_analysis/faf/> [Accessed 2025-01-15]
